# An exploratory study on the impact of the COVID-19 pandemic on adolescents’ psychosocial well-being in the Kwadukuza community

**DOI:** 10.11604/pamj.2026.53.29.49785

**Published:** 2026-01-25

**Authors:** Amanda Zuma, Raisuyah Bhagwan, Nomashodo Mirriam Siluma

**Affiliations:** 1 Department of Community Health Studies, Faculty of Child and Youth Care Programme, Durban University of Technology, Durban, KwaZulu-Natal 4000, South Africa

**Keywords:** Adolescents, COVID-19 pandemic, development, health, psychosocial well-being

## Abstract

**Introduction:**

the COVID-19 pandemic severely disrupted adolescents´ daily lives, affecting their psychosocial well-being. This study aimed to explore how the pandemic influenced mental health, social interactions, and coping strategies among adolescents in the KwaDukuza community, South Africa.

**Methods:**

a qualitative exploratory study was conducted using semi-structured interviews with sixteen adolescents selected through purposive sampling. Data were analyzed thematically using Braun and Clarke´s six-step framework to identify patterns in their experiences and coping mechanisms.

**Results:**

adolescents reported heightened stress, anxiety, depression, social isolation, and grief due to the loss of loved ones. Lockdown restrictions disrupted education and routines, while fear of infection and stigma were prevalent. Despite these challenges, participants demonstrated resilience through family support, spiritual practices, skill development, reading, and lifestyle adaptations.

**Conclusion:**

the study highlights the importance of family connectedness, community engagement, and culturally sensitive mental health interventions to strengthen adolescent resilience in under-resourced settings during public health crises.

## Introduction

South Africa, as a developing country, is characterized by many of its citizens being poor, with low income and holding hand-to-mouth jobs. The COVID-19 pandemic also led to thousands of people being retrenched from their jobs [1], which indirectly impacted the well-being of adolescents. Adolescents and their families from different backgrounds encountered various challenges, such as a lack of resources in households [2]. Whilst some adolescents acknowledged the resources which they had access to while they were at home, others acknowledged the prevalence of violence, hunger, and food insecurity, concern for their parents, siblings, and society at large during the pandemic.

Moreover, adolescents experienced great trauma when their family members became sick, and when the infected person was in isolation or quarantine [3]. Accordingly, this study sought to answer the research question: how did the COVID-19 pandemic affect adolescents´ psychosocial well-being in the KwaDukuza community? The source of worry emerged due to the uncertainty of being separated from the person who is sick, which caused adolescents to feel unsafe. Adolescents expressed great concern and fear about the health, safety, and well-being of their parents and siblings [4]. The lives of adolescents who were vulnerable were thus negatively impacted by these sudden changes, which caused further deterioration in their mental health. Although some adolescents comprehended the necessity of lockdown, they became further depressed as they would be stuck in one place for more than a week, which was unusual for them as young, developing individuals who were accustomed to socializing [5]. Being under lockdown illustrated persisting negative psychosocial impacts on adolescents due to the radical changes in their arrangements, physical activity, and overall lifestyle.

Asserted that knowledge about disasters is useful for helping people cope with the COVID-19 pandemic´s psychological and social consequences [6]. Many countries lack experience with catastrophes such as the COVID-19 pandemic and are often poorly equipped to handle other catastrophes adequately.

Psychosocial impairment is known to be influenced, but not limited to, the extent of injustice, poverty, social inequality, population growth, health standards, education levels, gender equality, and social capital [7]. According to the World Health Organization (2021), mental problems after disasters are expected to range from mild stress to very serious mental health problems. Adolescents are at greater risk, as a meta-analysis has shown clear developmental deficits after a disaster.

Adolescents were exposed to primary and secondary trauma during the pandemic. They suffered from burnout, daily stress, and emotional difficulties during the pandemic. The extent of post-traumatic symptoms appeared to be directly dependent on the perceived severity of the threat, quarantine experiences, and the number of people infected within the social network [8]. The pandemic obviously had negative psychosocial consequences, including stress depending on its type, social contexts, coping characteristics, and resilience factors.

The daily routines of adolescents were altered as their everyday lives were changed when the pandemic hit. As a result of the COVID-19 pandemic, adolescents experienced unprecedented interruptions to their daily lives [9]. This was the ideal precipitant of mental illnesses, such as stress-related symptoms, anxiety, depression, obsessive-compulsive disorder, and post-traumatic stress disorder. As the COVID-19 pandemic affected almost every individual´s life, adolescents experienced a range of emotions such as sadness, disappointment, anger, frustration, hopelessness, and loneliness [10]. There was little to no reason for adolescents to be happy as their lives changed, and some became vulnerable to the virus itself.

For many adolescents, the pre-existing recurring situations within the South African context heightened exposure to several risks during the COVID-19 pandemic. Research has suggested that adolescents firmly embed themselves within their social-ecologies. Adolescents expressed the need for a sense of belonging within their circle of friends, as opposed to their families and communities, some of whom may have previously rejected them [11]. During the pandemic, vulnerable adolescents were unable to access various individual internal resource systems and reach out to obtain support from others. They had to draw on a range of internal resources to help them cope with the assistance of caregivers, some of whom provided therapeutic support as well.

As access to resources was very limited during the lockdown, adolescents had to resort to what was readily accessible to them. Many adolescents acknowledged the protection and support provided to them, which made it easier for them to cope. Being within a familial context was of great importance for the overall well-being of adolescents, along with having their basic needs met, such as food, shelter, and safety [12]. The home context of adolescents was found to be essential to the provision of an environment that was stable, consistent, and that which was built on supportive relationships. This also applies to their social relationships.

This research study is therefore timeous and valuable as limited research was undertaken related to the psychosocial well-being of adolescents during the COVID-19 pandemic in South Africa and abroad [13]. The purpose of the study was to understand the psychosocial effects of the COVID-19 pandemic and to understand how the psychosocial well-being of adolescents could be strengthened during the pandemic [14]. The strategies that were to be developed were intended to assist adolescents not only during the pandemic but also in the future, where they could use established ways of coping and be better able to respond appropriately to a situation similar to the COVID-19 pandemic. In this study, mental health refers to emotional states such as stress, anxiety, and depression; social isolation denotes reduced social interactions and limited peer contact due to lockdown; and coping mechanisms describe strategies individual or collective used by adolescents to manage pandemic-related challenges.

**Literature review:** the COVID-19 pandemic, along with its containment measures, caused increased concerns for the overall well-being, social life, mental and emotional health of adolescents and their families. For adolescents, the confinement was accompanied by feelings of worry, fear, sadness, hopelessness, discomfort, anger, frustration, and boredom with having to be away from school and peers [15]. Even though this was a crucial time for families to strengthen their bonds and support one another, given the hardships that came with the pandemic, adolescents had needs that were unmet.

Adolescents were engulfed by many changes during the duration of quarantine. This was accompanied by a restriction on personal freedom and their social life. Adolescence is a critical developmental stage in which a sense of autonomy must be protected; hence, the restriction and deprivation of freedom should not go unnoticed [16]. During adolescence, changes lead to a perception of risk, causing thoughts that inevitably lead to harm. Hence, adolescents tend to be more exposed to risky behavior. Increased predicted conflicts with authority and resistance in abiding by rules were not unforeseen, as it was difficult for adolescents to perceive the hazards caused by the COVID-19 virus and to be able to comply with protective and preventive measures [17]. Research findings within the literature revealed that compliance with precautions was difficult for adolescents compared to adults. Anxiety, mood swings, and self-harming ideation further manifested amongst adolescents within the family nucleus.

Adolescents experienced frustration, loneliness, anxiety, irritability, despair, depression, denial, and obsessive-compulsive disorder symptoms as a result of the global pandemic. Furthermore, adolescents with pre-existing severe mental health illnesses experienced more intense discomfort [18]. With the increased threat of deterioration, consideration of the psychosocial well-being of adolescents became more critical. Moreover, adolescents who required therapy experienced disruption in terms of access to mental health services and medication, thereby causing an impairment of emotion regulation and adaptive coping [19]. The pandemic´s impact on psychosocial health had huge mental health sequelae which suggests its detrimental effects on their mental health. While prior studies identified adolescent stress and anxiety during the pandemic, many relied on cross-sectional surveys with limited qualitative depth. Few examined adolescents´ own narratives in low-resource contexts, leaving a gap that this study addresses.

### Theoretical framework

**Erikson´s psychosocial development theory:** this study was grounded in Erikson´s psychosocial development theory, which provides a developmental lens for understanding how pandemic-related disruptions affected adolescents´ sense of identity, competence, and psychosocial growth. Erik Erikson identified eight developmental stages, each defined by a psychosocial challenge that must be successfully resolved to ensure balanced personality growth. Each stage builds upon the successful completion of previous ones, and difficulties encountered earlier may re-emerge as psychological challenges in later life [20].

During adolescence, individuals face the identity versus role confusion crisis-a period marked by exploration, experimentation, and the search for personal coherence. Role confusion can arise when external disruptions, such as social isolation and loss of daily structure during the COVID-19 pandemic, interfere with this developmental process [21]. Erikson emphasized that psychosocial crises result from the interaction between individual needs and social expectations, and their resolution determines one´s ability to develop self-esteem, independence, and meaningful social connections [22].

Adolescents rely on peer interaction, achievement experiences, and opportunities to demonstrate competence to navigate this stage successfully. However, pandemic restrictions disrupted schooling, friendships, and community participation, leading to uncertainty, reduced confidence, and a diminished sense of purpose. These challenges reflect Erikson´s notion that unresolved crises can hinder the development of identity and autonomy. For adolescents in KwaDukuza, lockdowns curtailed essential developmental experiences such as teamwork, socialization, and skill-building that are critical for forming a stable sense of self [23].

Erikson´s framework therefore offers a valuable interpretive lens to understand how the COVID-19 pandemic interrupted developmental milestones and contributed to psychosocial distress. It also highlights the importance of protective systems particularly family support, community belonging, and emotional guidance in rebuilding self-efficacy and resilience when external stressors threaten developmental progression [24].

## Methods

**Study design:** this study adopted a qualitative exploratory design, which is appropriate for gaining in-depth insights into participants´ lived experiences and perceptions. A qualitative approach was selected because it captures the depth, complexity, and meaning of adolescents´ lived experiences, dimensions that cannot be easily quantified through surveys. This study adopted a qualitative exploratory design, which is appropriate for gaining in-depth insights into participants´ lived experiences and perceptions. Qualitative research allows for rich, descriptive exploration of social and emotional phenomena that cannot be quantified. This design enabled the researchers to explore adolescents´ psychosocial experiences during the COVID-19 pandemic and to understand the coping mechanisms they employed within their community context.

**Study setting:** the study was conducted in KwaDukuza, a semi-rural community located in KwaZulu-Natal Province, South Africa. KwaDukuza is characterized by socio-economic diversity, with sections of informal settlements and limited access to social services. The area was significantly affected by the economic and psychosocial consequences of the COVID-19 pandemic, including unemployment, school closures, and restrictions on movement. Data collection took place between June and September 2022, when COVID-19 restrictions in South Africa had been partially lifted, but the psychosocial effects remained evident among communities.

**Population and sample:** the study targeted adolescents aged 14 to 18 years residing in the KwaDukuza community during the COVID-19 lockdown period. This developmental stage was selected because adolescence is a critical phase marked by intense psychosocial and emotional transitions, rendering individuals more vulnerable to external stressors such as pandemics and social restrictions. A non-probability purposive sampling technique was employed to identify participants who met the inclusion criteria. Adolescents were considered eligible if they: 1) were between 14 and 18 years of age; 2) had resided in KwaDukuza throughout the national lockdown; 3) were willing and able to provide informed assent, supported by parental consent; and 4) could communicate effectively in either English or isiZulu. Those with severe cognitive or emotional impairments that could interfere with communication or informed participation were excluded.

The ward councillor assisted the researcher in locating households with eligible adolescents. Initially, twelve participants (six males and six females) were recruited. However, recruitment continued until data saturation was achieved, defined as the point at which no new codes or themes emerged from the interviews. Saturation was reached after sixteen interviews, ensuring both thematic depth and diversity of experience within the dataset. This final sample size was deemed adequate for qualitative analysis, allowing a comprehensive exploration of adolescents´ psychosocial well-being during the COVID-19 pandemic. The use of the ward councillor to identify households may have introduced selection bias by favoring more visible or accessible families; however, efforts were made to include adolescents from diverse residential zones and socioeconomic levels.

**Data collection tools and procedures:** data were collected using a semi-structured interview guide, which had been pre-tested to ensure clarity and relevance. The interview guide was pre-tested with two adolescents from a nearby community to assess clarity, sensitivity, and cultural appropriateness; feedback informed minor wording revisions. The guide included open-ended questions focused on: adolescents´ experiences during the pandemic; perceived effects on their mental and social well-being; coping strategies used; and sources of family or community support.

Before each interview, the researcher explained the purpose and procedures of the study to participants and their guardians. Informed consent was obtained from parents or guardians, while assent was obtained from each adolescent participant. Interviews were conducted individually in a quiet and private community hall setting to ensure confidentiality and comfort. Each session lasted between 30 and 45 minutes. With participants´ permission, interviews were audio-recorded and supplemented by field notes documenting non-verbal cues and contextual observations. Interviews were conducted primarily in English and isiZulu, depending on the participant´s preference, and later translated and transcribed verbatim into English.

**Data analysis:** data were analyzed using thematic analysis, following the six-step framework proposed by Braun and Clarke: 1) familiarization: the researcher repeatedly read transcripts to immerse in the data; 2) coding: meaningful phrases were highlighted and manually coded to identify recurring concepts; 3) theme development: codes were grouped into potential themes and subthemes; 4) reviewing themes: themes were refined for internal coherence and distinctiveness; 5) defining and naming themes: each theme was clearly defined and supported with participant quotations; 6) reporting: themes were integrated into a coherent narrative reflecting adolescents´ experiences and coping patterns.

A manual color-coding technique was employed for thematic grouping. Peer debriefing was conducted with co-authors to validate coding consistency and interpretive accuracy. Any discrepancies were discussed until a consensus was reached. A sample coding matrix illustrating the link between initial codes, subthemes, and final themes is presented in Table 1 to enhance transparency of the analytic process (Table 1). Any discrepancies were discussed until a consensus was reached. A sample coding matrix illustrating the link between initial codes, subthemes, and final themes is presented in Table 1 to enhance transparency of the analytic process. Data were analyzed manually without qualitative software (e.g., NVivo) to maintain direct engagement with the text during coding.

**Table 1:** example of coding matrix linking codes, subthemes, and themes

Initial codes	Subthemes	Themes
“Felt trapped indoors,” “missed seeing friends,” “restricted movement”	Disruption to daily routines	Mental health well-being
“Couldn’t attend funerals,” “felt unsafe,” “no proper send-off”	Inability to mourn or grieve	Loss of lives
“Prayed with family,” “read Bible for comfort”	Prayer and spirituality	Coping strategies

**Trustworthiness:** in order to achieve credibility, the researcher ensured an understanding of the research participants and notes that a description of the context and processes was as accurate and complete as possible and that the interpretations are inclusive. Confirmability is an audit trail where the researcher records all the steps taken and the decisions made regarding the data coding and analysis for rationalization purposes. Transferability refers to the extent to which the researcher´s interpretation or conclusions are transferable to other similar contexts. This required a thorough and in-depth description of the research activities and assumptions as was followed. Although findings are specific to KwaDukuza, lessons drawn, such as the role of family support and spirituality, may inform psychosocial support programs in other under-resourced communities facing public health emergencies.

**Ethical considerations:** ethical clearance for the study was obtained from the Durban University of Technology Institutional Research Ethics Committee (Ref. No: DUT-CHS-REC-2022/018). Permission to conduct the study was also granted by the KwaDukuza ward councillor.

All participants and guardians were informed of their right to withdraw at any stage without penalty. To maintain confidentiality, pseudonyms were used, and all identifying information was removed from transcripts. Audio files and transcripts were securely stored in password-protected digital folders accessible only to the research team.

**Study limitations:** as a qualitative study, findings are context-specific and may not be generalizable beyond KwaDukuza. Additionally, interviews relied on self-reported experiences, which may be influenced by recall bias or social desirability. However, rigorous methods of verification and triangulation enhanced the credibility of findings. However, rigorous methods of verification and triangulation enhanced the credibility of findings. Potential interviewer bias and translation effects may have influenced interpretations, though cross-checking of isiZulu-English transcripts and peer debriefing helped mitigate these limitations.

## Results

**Theme 1: mental health well-being:** the first theme that emerged was mental well-being. There were five subthemes that emerged from the data, i.e., stress, anxiety, and depression; disruption to daily routine and hopes and dreams; effects of the lockdown regulations; fear of being infected; and stigma and discrimination.

**Sub-theme 1: stress, anxiety, and depression:** participants described their stress, anxiety, and depression as follows: *“The lockdown was very stressful and being stuck in one place having nothing to do. It was a horrible experience. People kept to themselves mostly at home, so I had to find other ways to entertain myself to avoid being depressed. Even though we couldn´t do what we preferred, I had to find something to pass the time by*” P14. *“That caused bad anxiety and uncertainty; I had to move back home, and I stayed with my aunt for school purposes. My mental health has not recovered since then because I could not get used to the situation”* P8.

**Sub-theme 2: disruption to daily routine and hopes and dreams:** participants said that the COVID-19 pandemic caused disruptions to daily routines and hopes and dreams as follows: *“I was very sad and frustrated that I was not able to do things that I wanted and I felt trapped most of the time, so what I used to do was to take deep breaths and also tried to relax when I felt like I was exploding with thoughts. It was a terrible experience as it ruined my plans for the year. Due to the restrictions, I could not go ahead with my driving course, which was going to be during the school holidays, and I also lost a part-time job that would have earned me extra cash. It was challenging to move around and there were limited things to do”* P6.

*“Adjusting to the COVID-19 restrictions is the most challenging factor. Changing my daily routine was a bigger problem, as was a sudden change in my diet, as I couldn´t eat what I wanted to, and not being where I wanted to be when I wanted to was another challenging thing. The long wait in the queue made shopping for groceries unbearable because they wanted a small group of people inside the shops. Hospitals did not allow visitors, and we were denied time to be with our sick loved ones. The thought of wearing a mask whenever you go out, while you felt suffocated, caused additional stress, fear, and anxiety”* P10. *“I´m an outgoing person, so the isolation was very difficult as I was not able to go shopping nor anywhere, as movements required permits and only the essential workers´ movements were allowed, e.g., shopping or medical necessities. We had to stay indoors”* P3.

**Sub-theme 3: effects of the lockdown regulations:** the third sub-theme focused on how the lockdown regulations during the COVID-19 pandemic disrupted adolescents´ everyday lives. Rapidly increasing infection rates led the government to enforce strict restrictions, which significantly limited mobility and social interaction. Participants described the experience as follows: *“it was a hectic experience because I had to be indoors 24/7. I couldn´t attend soccer practice or visit friends, and even grocery trips were restricted to certain times. Essential needs were the only ones we could access”* (P15).

*“Only essential shops were open, and we couldn´t go out easily. I had panic attacks from constantly being indoors and worrying about touching contaminated surfaces”* (P8). *”Overall, participants expressed that lockdown regulations created a sense of confinement and anxiety, as their normal routines and social freedoms were suddenly taken away”* P5.

**Sub-theme 4: fear of being infected (personally and loved ones):** the fourth sub-theme explored participants´ fears of contracting COVID-19 and concerns for the health of their loved ones, particularly elderly or chronically ill family members. Participants expressed: *“I was frightened for my dad, who had a chronic illness, so we had to be more vigilant at home and follow safety precautions strictly”* (P16). *“When I started developing flu-like symptoms, I panicked because the news said those were signs of the virus. The more I thought about it, the more I felt the symptoms”* (P13). *“Overall, participants described constant anxiety and hypervigilance, fearing both personal infection and the possibility of transmitting the virus to vulnerable family members”* P4.

**Sub-theme 5: stigma and discrimination:** the fifth sub-theme derived from the data was focused on stigma and discrimination. Participants said as follows: *“there was a stigma as those who had been infected with the virus were looked at differently”* P3. *“I felt as if we also discriminated against one another when one would be infected, for example, my uncle began to have a fever and felt a sore throat. He caught a cold because he was used to going outside to sit with people, while he knew very well that there was a deadly virus at large. So, I felt that there was segregation within the family. There was also segregation in relationships as there were no gatherings and I felt that it was going to be hard to rebuild relationships as we did not know when the COVID-19 pandemic would end”* P4.

**Theme 2: social isolation:** the second sub-theme that emerged from the data was social isolation. This theme included two sub-themes, i.e., loneliness and maintaining friendships.

**Sub-theme 1: loneliness:** the first sub-theme focused on loneliness. The participants said as follows: *“for the first time in my life, I felt like I was in prison. Not being able to leave the house was unsettling and made life very difficult as I felt the need to be with my peers. Furthermore, I couldn´t hang out with my friends to socialize”* P6. *“I was very lonely as I couldn´t see my friends, and I was trapped at home. The isolation process of being indoors all the time scared me and I thought that it was the end of the world. I also couldn´t go to church to praise as I knew that even if I did not go to school that day, I would see my friends on a Sunday, regularly”* P4.

**Sub-theme 2: maintaining friendships:** The second sub-theme that emerged from the data was maintaining friendships. *“My friends and I contacted one another by means of phone calls, more especially video calls, as they made communicating better as you were able to see one another. Although it was difficult to reach others as the network was very bad most of the time, we could only place calls when cellular connectivity allowed us to”* P6. *“I kept in touch with my friends using my cell phone by calls and SMSing, and also through social media. It played a huge role in keeping me sane because I needed people to talk to as I was the only child at home”* P15.

**Theme 3: family connectedness:** the third theme focused on family connectedness. Two sub-themes emerged from the data, i.e., strengthening of good family relationships and shared family activities.

**Sub-theme 1: strengthening of good family relationships:** the first sub-theme focused on strengthening good family relationships. *“It was good to spend more time with my family, and I found things that also made me happy to do with my family. This was not usual as my mother worked in the city of Durban and did not live with me. We had more family time, watched the news almost 3 times a day and always listened to what the president would say”* P7. *“My mother did the gardening work, and I helped her. We also cleaned together, cooked meals, and also prayed together as a family all the time. We couldn´t sleep and had nothing to do during the day, so that helped us spend more time together. My mother worked in another town, 2 hours away from our home, so she was usually away at her job. During the COVID-19 pandemic, she was relieved of her job and came back home, so we spent a lot of time together with her and other family members and got to know one another thoroughly”* P3.

*“There was improved communication within families as they got to talk to one another every day and everyone was concerned for one another as we always checked up on each other”* P5. *“We got to spend more time together as a family during the day doing activities collectively, especially after the passing away of my father. I did not allow the pandemic to get over my head and make me forget the people close to my heart. From time to time, I would give them reassurance that there is life ahead and we need to be strong to move on”* P10. *“We would phone other family members to find out how they were doing and also bonded with the family most times”* P7.

**Sub-theme 2: shared family activities:** the second sub-theme focused on shared family activities. *“During the pandemic, since a lot was restricted, it is when I got to spend more time with my family. To strengthen our relationship, we started doing indoor activities together, such as exercising, dancing, playing video games for enjoyment purposes, and redecorating the house. The activities that we did together as a family helped us strengthen relations amongst one another since we got to learn more about each other”* P11. *“I spent time with my siblings and parents to catch up with them. This benefited me as it was a good experience, and it enhanced our co-existence. We had to compromise with one another by sharing things at home, like the TV, phones, games, as well as information. This was unusual because I was away from home most of the time. I was not used to being around many people anymore”* P8.

**Theme 4: loss of lives:** loss of lives emerged as the fourth theme. This included two sub-themes the trauma of losing loved ones and the inability to mourn and grieve.

**Sub-theme 1: the trauma of losing loved ones:** the first sub-theme focused on the trauma of losing loved ones. *“Most people close to me were infected, and I lost someone close to me. I was very close to the person who passed away, as I had a birthday which they came to, and a week later, I lost the person. Everything happened so fast”* P9. *“It was so unfortunate that we had to lose my dad, who was the breadwinner of the family. Some of the family members were unable to attend his burial. The thought of him lying in hospital and not getting the chance to see him till his last days are wounds we could never heal from”* P10.

**Sub-theme 2: inability to mourn or grieve:** the second sub-theme focused on the inability to mourn or grieve. *“We couldn´t meet relatives or even pay our respects to those who have lost their loved ones and who were close to us. I felt so unsafe which was unpleasant”* P13. *“I was unable to accept all of it at once as they couldn´t even get a proper send-off. This was rather emotional for me and traumatic as well because we had to bury them like an animal. It felt as though we were throwing them away just like that”* P17. *“We waited for the restrictions to be lifted to do rituals which should have accompanied the burial”* P9.

**Theme 5: lifestyle changes:** the fifth main theme was lifestyle changes. Two sub-themes emerged from the data, i.e., transition from contact lessons to online lessons and financial distress endured by families.

**Sub-theme 1: transition from contact lessons to online lessons:** this sub-theme focused on the transition of contact lessons to online lessons. Participants said as follows: *“it was very hard as I couldn´t interact with the teacher and other learners from school as we previously did before. We had to understand and apply things learnt digitally, as this required internet connectivity for a long period, which was a problem to afford at times. I preferred contact lessons as it was practical and teachers were able to elaborate more when in front of the class, as opposed to online learning where there were network issues and teachers cruised through the work”* P16.

*“Online learning was more appropriate for me, although there was more work to be done, but I managed. Sometimes I felt under pressure to finish the work. It was not easy to do it online as I had never done it before. I also had network problems when I had to research some things for schoolwork. There were complications using gadgets such as laptops and phones, as I was not used to using them. I just knew the basics. I had nobody to help me with schoolwork because my parents did not reach high school level”* P6 (female). *“I couldn´t even access the library for other relevant resources. There were also network issues and interruptions at home during the day. Online learning affected my progress as there were more interruptions when I tried to focus, and I preferred contact lessons. I also had issues with data and there were also interruptions on the phone as well but I had to adapt”* P13. *“I did the work just because I had to do it, but I did not enjoy it because before I used to do my homework at school to avoid having to work at home. There was poor connectivity of the internet and interruptions by family members during the day, so I had to do my schoolwork at night. Sometimes my older sister, who was in university, helped me with my work. It was very difficult to understand some of the content that was taught. Learning became too much, and it became a drag”* P12.

**Sub-theme 2: financial distress endured by families:** the second sub-theme focused on the distress families endured due to financial difficulties. Participants said as follows: *“during the COVID-19 pandemic, the food prices increased extensively, and we had to cut down on purchasing more meat to make food last the whole month. Life then became more expensive as it seemed we would soon not be able to afford to buy food and there was less money that was coming into our household”* P3. *“We suffered to afford a livelihood at home as there was no longer a basic income in the family, as my mother had lost her job. I was also worried about the health of my parents as well as my own, daily. Given that my mother had to pay for everything in our household, I would sell sweets at school for extra pocket money as I felt the need to assist with buying bread at home, so my business was also disrupted and it was very challenging to sell to people in the community as most of them were afraid to go out and were concerned about contracting the virus. So financial issues became more troubling for us. My dad was self-employed as a plumber, and he couldn´t work as he did not have any customers. The money that we had was only available for food, and we had to spend it wisely”* P7.

*“We had to rearrange the budget to meet our basic needs in the household because my mother was the only one who received an income. My brother had his salary reduced because he didn´t go to work regularly, so we experienced a massive financial strain at home. Fortunately, my dad had savings, so we used them for household utilities”* P16. *“My mother established a business and started making face masks for us to get extra cash to have more food. We took the opportunity to sell what we could at the time”* P9.

**Theme 6: positive effects of the pandemic:** the sixth theme focused on the positive effects of the pandemic. Participants said as follows: *“I also started a business during level 4 and fried chips. This kept me busy and also gave me extra money. At first, people were scared to come buy, but those who were close to me proved to the other people in the community that I was very hygienic when preparing the food. The business helped keep my mind focused as I was also thinking about other business ideas. I was able to view life differently, and I also wanted to be self-employed in the future. I was also more interested in the current affairs and followed the news channels”* P13. *“I spent most of my time browsing online. I learnt to farm, whereby I planted vegetables, and I started a business by selling them in the community. I invested most of my time in it, and I used to run daily operations. I learnt new skills and improved my technical skills on the phone, where I promoted my business via TikTok because I spent time on the internet to know what was happening around the world. After all, physical contact was prohibited”* P10.

**Theme 7: coping strategies:** the seventh theme focused on strategies used to cope with adversities during the pandemic. There were four sub-themes that emerged, i.e., familial support, prayer and spirituality, reading, and dietary changes.

**Sub-theme 1: familial support:** sub-theme 1 focuses on familial support. Participants said as follows: *“COVID-19 made me realize the importance of actually appreciating the things and people that we took for granted. What it taught me the most is the spirit of togetherness by always being supportive to your family and friends during both difficult and happy times, and helping one another when we still could. It was emotionally draining having to lose thousands of people”* P10. *“Learning to live with what you have and realizing how short life was as many people were dying in a short period, so I had to become more conscious about loving my family members when they were still alive, and help for the future if we ever survived. I also got to see who cares the most about our family. Most support proved that people were concerned about us”* P4. *“As a family, we spent time together mostly with one another to comfort each other by sharing joyful memories of the past and remembering the good that we had achieved as a family, not only focusing on negative thoughts. After the restrictions were lifted, we went out for lunch and had some fresh air as we felt like home was very toxic at that time. I was hoping for more settling experiences so that I could move on with life even though it was very hard”* P9.

**Sub-theme 2: prayer and spirituality:** the second sub-theme that emerged was prayer and spirituality. The participants said as follows: *“prayer helped a lot through the hard times as I was hopeful for a better future without restrictions, even though we were still in that situation. We had hoped and prayed in the hardest times to keep us sane, and we felt that God would intervene at some point. My family is spiritually strong, and we also have a strong belief in ancestors, so much so that we didn´t abandon our customs at the time of the pandemic. We continued to burn some incense as well to request that our forefathers remain with us and protect us during that difficult period”* P1. *“We prayed all the time. I also read the Bible as my mother used to read a verse every day. Church was closed so I also listened to gospel songs during the day which helped me to calm down when we were not watching the news”* P7.

**Sub-theme 3: reading:** the third sub-theme that emerged was reading. One participant said as follows: *“I also read novels which were in the house that belonged to my late father, repeating them as I had all the time in the world. I also browsed through the internet because my big brother lent me his phone sometimes, but it was not such a good idea to be on the internet, as it had stressful news all the time. This way, I was able to escape the reality of the pandemic and avoid negative thoughts. It was also a form of a coping mechanism for me and passing the time. Sometimes I felt like being alone and interactions were not ideal for me”* P12.

**Sub-theme 4: dietary changes:** the fourth sub-theme that emerged was dietary changes. One participant said as follows: *“I learnt how to cook and bake as cooking was very therapeutic in terms of relaxing my mind and eliminating negative thoughts of illness and death. So, I explored new dishes for my family. This uplifted my soul and inspired my mood and spirit. It also promoted my positive self-esteem, and it was able to keep me distracted away from negative thinking”* P9 (Table 2).

**Table 2:** summary of main themes and subthemes identified in the study

Theme	Subthemes	Representative quotes	Key insights
Mental health well-being	Stress, anxiety, and depression; disruption to daily routines; fear of infection; stigma	*"The lockdown was very stressful… I had to find other ways to entertain myself"* (P14)	Adolescents experienced psychological strain from confinement, uncertainty, and health fears
Social isolation	Loneliness; maintaining friendships	*"For the first time in my life, I felt like I was in prison"* (P6)	Lack of peer contact and school closures increased loneliness, though digital tools helped maintain some connections
Family connectedness	Strengthening family relationships; shared family activities	*"It was good to spend more time with my family… we cooked, prayed, and watched the news together"* (P7)	Family time improved emotional support and strengthened relationships during lockdown
Loss of lives	Trauma of losing loved ones; inability to mourn or grieve	*"We couldn’t even pay our respects… I felt so unsafe"* (P13)	Restrictions disrupted mourning processes, causing emotional distress and unresolved grief
Life>	Transition to online lessons; financial distress	*"Online learning was hard… poor connectivity and distractions at home"* (P16)	Adolescents faced challenges adapting to online education and household financial pressures
Positive effects	Skill-building; entrepreneurship	*"I started a business during Level 4 and fried chips… it kept me busy"* (P13)	Some adolescents developed resilience through creativity, learning, and entrepreneurship
Coping strategies	Family support; prayer and spirituality; reading; dietary changes	*"Prayer helped a lot through the hard times… we prayed all the time"* (P1)	Coping mechanisms included spirituality, family bonding, and constructive personal routines

## Discussion

The pandemic increased uncertainty about the future, leaving adolescents wondering what was next. A sudden change created doubts about whether life would ever normalize. According to children and adolescents were likely to experience higher rates of depression and anxiety during this period [25]. Therefore, effective mental health services were essential to reduce these rates and improve their well-being. Adolescents could not develop social skills, empathy, or a sense of identity during this time due to deprivation of social interactions. The disconnection from social settings during the lockdown led to a loss of peer and social outlet connections, as reflected in their narratives. Researchers such as Ajanovic *et al*. [26] further noted that adolescents experienced setbacks not only academically but also emotionally and socially.

Restrictions related to COVID-19 impacted adolescents´ mental health. Existing studies show that those with pre-existing mental health issues experienced further increases in depression and anxiety, indicating a decline in their ability to adjust [27]. Feelings of loss, heightened emotions, and routine disruptions due to lockdown mainly contributed to increased anxiety, with fewer positive experiences encountered. The restrictions aimed at reducing infection rates disrupted daily routines for adolescents and their families, prompting unfamiliar changes. Prolonged school closures, for instance, forced rapid adjustments in diets, extracurricular activities, outdoor pursuits, and social organization. The abrupt withdrawal from school led to increased anxiety [28]. When infection rates rose, schools closed, and strict home confinement was imposed, restricting adolescents´ movements to essential activities only [29]. Limited mobility and social interactions heightened feelings of confinement.

The pandemic created extraordinary circumstances, leading to life changes due to measures like lockdowns [30]. These restrictions significantly reduced outdoor activity and physical movement, requiring individuals to adjust their schedules [31]. Community members mostly stayed indoors, leaving only for emergencies such as medical needs or procuring essentials. High infection rates fostered feelings of insecurity among adolescents and their families. Concerns intensified when loved ones showed flu-like symptoms, raising fears of infection.

Adolescents experienced anxiety about the potential death of parents or guardians, adding financial strain to families [32]. Parents worried about surviving the pandemic and the risk of losing their entire family, which led to increased safety precautions to minimize contact and exposure. Many feared hospitalizations; some preferred home remedies to avoid hospital admission due to fears of death and restrictive visitation policies. The death of patients also caused emotional distress at home, and restrictions on visitors in hospitals further complicated adolescents´ concerns about family members´ visits.

COVID-19-related stigma was evident, with perceptions spreading that a cough or sneeze indicated infection, leading to avoidance, rumors, and social breakdowns. Social restrictions caused adolescents to lose face-to-face interactions, resulting in loneliness and emotional distress [33]. Spending more time with family and less with peers altered socialization patterns, which are vital during adolescence. School closures, the central social context, deeply impacted peer relationships, causing distress from social isolation and feelings of loss. While online platforms helped maintain some social contact, they could not fully replace in-person interactions, leaving adolescents vulnerable to depression and loneliness. Technology, however, aided communication and provided emotional relief, fostering hope and connection.

Family activities during lockdown strengthened bonds and supported adolescents´ emotional, social, language, and cognitive development. Many families engaged in indoor games and conversations to combat boredom, sadness, and anxiety caused by school closures and quarantine. These shared activities fostered reassurance and optimism for the future. However, increased mortality, often sudden, disrupted normal grieving processes due to restrictions on hospital visits and funerals. Adolescents had questions about family deaths, and families had to deliver difficult information calmly and clearly [34]. The rapid and unanticipated nature of COVID-19 deaths meant that mourning was often incomplete or trivialized, leading to disenfranchised grief manifesting as rage, shock, guilt, disbelief, and other intense emotions compounded by the inability to grieve properly. Funerals were rushed, raising questions about burial procedures and preventing proper grieving, often leaving families with feelings of helplessness.

The economic impact was profound, with job losses and financial hardship causing parents to lash out at children. Daily routines changed, and the lack of outdoor activities and social support heightened uncertainty for both adults and children [35]. Limited access to technology further disrupted education, especially in low-income households lacking devices or reliable internet, and increased household noise and distractions hindered learning. Many families faced poverty due to reduced income and job insecurity, disproportionately affecting those with the lowest incomes.

Despite these hardships, some adolescents found opportunities for growth. They demonstrated resilience by devising new ways to earn income, enhancing their skills, and assuming leadership roles using digital platforms to promote talents like dancing, drawing, singing, and artworks [36]. Digital technology enabled them to develop their businesses and foster innovation, contributing to their personal development and long-term sustainability.

Parents also worked to strengthen emotional resilience, engaging more actively with their children and supporting their responsibilities at home. The shared effort helped maintain family harmony and fostered empathy. Participants reported experiencing significant adversities and adjusting rapidly to emergency measures to stay safe. Many had pre-existing challenges like poverty, depression, and educational disruptions, which were further aggravated during the pandemic [37]. The abrupt transition to restricted routines caused concern about the future and triggered negative emotions, including paranoia and fear of the unknown.

Regarding psychosocial well-being, adolescents faced psychological, social, and emotional difficulties. They experienced decreased positive emotions, hopelessness, social isolation, loneliness, and a diminished sense of control. Changes in living arrangements and family roles increased familial conflicts and reduced communication, especially among vulnerable and lower-income groups. Nonetheless, adolescents employed coping strategies such as family support, prayer, spirituality, reading, dietary changes, and physical activity [38]. They used positive thinking, cognitive reappraisal, and secondary control to manage stress, with families fostering resilience through positive parenting and emotional support.

Spiritual practices like prayer and singing provided outlets for expressing feelings, fostering hope, and maintaining motivation. Reading increased during the pandemic and was associated with improved skills. Lifestyle adjustments, including healthier diets, also helped adolescents cope with stress [39]. These strategies underscored the resilience of adolescents and their families during this challenging period.

## Conclusion

This study explored the psychosocial well-being of adolescents in KwaDukuza during the COVID-19 pandemic, focusing on their emotional challenges and coping strategies. The findings revealed that adolescents experienced heightened stress, anxiety, and social isolation due to school closures, movement restrictions, and fear of infection. However, many demonstrated remarkable resilience by engaging in adaptive activities such as strengthening family bonds, exploring creative skills, and drawing comfort from spirituality and prayer. The study underscores the crucial role of families and communities in supporting adolescents´ mental and emotional health during crises. Continuous emotional and psychosocial support is essential for fostering self-esteem, confidence, and overall well-being among youth, particularly in under-resourced communities. Strengthening community-based support systems and integrating culturally sensitive mental health interventions can help mitigate the long-term effects of pandemics on young populations. Future research should examine how these coping strategies can be implemented and scaled at community and institutional levels, and assess the role of social service professionals in rebuilding family and community life in post-crisis contexts. Practically, interventions could include youth mental-health clubs, community counselling programs, and partnerships with schools and faith-based organizations to deliver psychosocial support during future crises.

### What is known about this topic

- Adolescents are highly vulnerable to psychosocial challenges such as anxiety, depression, and social isolation during public health crises like the COVID-19 pandemic;
- Disruptions to school, social life, and daily routines negatively affect adolescents´ mental and emotional well-being;
- Coping strategies involving family support, spirituality, and social connectivity help buffer the psychological effects of confinement and uncertainty.

### What this study adds

- The study demonstrates that the pandemic also created opportunities for strengthened family relationships and shared household activities that enhanced adolescent resilience;
- It identifies practical coping mechanisms, including skill-building, small-scale entrepreneurship, and lifestyle adaptations that promote positive adjustment among adolescents;
- It emphasizes the importance of community-based and culturally sensitive mental health interventions to support adolescents in resource-limited settings during and after major crises.
